# A Rare Combination of Chromosomal Abnormalities in an Infant With Turner Syndrome and Hypoplastic Left Heart Syndrome

**DOI:** 10.7759/cureus.16500

**Published:** 2021-07-20

**Authors:** Roya Huseynova, Latifa Bin Mahmoud, Abduljabbbar Alshenqiti, Khalid Alomran, Nabeel Alodaidan, Ogtay Huseynov

**Affiliations:** 1 Neonatal Intensive Care Unit, King Saud Medical City, Riyadh, SAU; 2 Genetic and Metabolic Unit, King Saud Medical City, Riyadh, SAU; 3 Cardiology, King Saud Medical City, Riyadh, SAU; 4 Neurosurgery, Azerbaijan Medical University, Baku, AZE

**Keywords:** hypoplastic left heart syndrome, translocation, neonates, turner syndrome, 14q11.2 microduplication syndrome

## Abstract

Hypoplastic left heart syndrome (HLHS) is a fatal congenital complex heart defect where the heart's left side is critically undeveloped. However, its pathogenesis remains unknown. We report a unique case of HLHS because of the rare combination of two abnormalities in the cell lines: partial monosomy X (Turner syndrome) and partial trisomy 14 (14q11.2 microduplication syndrome).

## Introduction

Hypoplastic left heart syndrome (HLHS) is a congenital heart defect constituting 2% to 9% of all congenital heart diseases [1]. The mortality rate accounts for 25% of neonatal cardiac deaths [2,3]. This cardiac structural defect includes varying degrees of the left ventricle's underdevelopment, hypoplasia of the aorta, aortic valve and mitral valve stenosis, or atresia [2]. The management options of HLHS include comfort care, surgical-staged Norwood surgery, and cardiac transplantation.

The etiology of HLHS is multifactorial and includes maternal, infectious, immunosuppressive, and genetic factors [4]. Although there is growing data which are supporting a genetic etiology, no specific gene has been identified until now [5-7]. Some reports have concluded that HLHS is heterogeneous in etiology [8]. The outcome in HLHS is significant influenced by the presence of chromosomal and other noncardiac abnormalities.

We present a unique case of HLHS because of the rare combination of two abnormalities in the cell lines: partial monosomy X (Turner syndrome) and partial trisomy 14 (14q11.2 microduplication syndrome).

## Case presentation

A term girl, small for gestational age, the first baby of the first degree consanguineous couple, was born to a 29-year-old mother by spontaneous vaginal delivery. The pregnancy was insignificant, and there was no history of radiation exposure or drug intake in any trimester; no family history of congenital heart disease or any chromosomal disorders.

A prenatal ultrasound scan at 30 weeks of gestation revealed a hypoplastic left ventricle defect, with no extracardiac malformations. Fetal echocardiography reported hypoplastic left heart, atrial septal defect, and patent ductus arteriosus. Amniocentesis was done at 32 weeks of gestational age, and chromosomal microarray analysis (CMA) revealed partial monosomy and partial trisomy. 

An approximately 63 megabases large pathogenic one copy loss (heterozygous deletion) encompassing the entire small arm of the chromosome X (Xp) and extending to the long arm of chromosome X (Xq), X p22.33q11.2 arr[GRCh37] Xp22.33q11.2(168546_63625851)x1) containing 447genes was detected. Furthermore, an approximately 50 megabases large pathogenic one copy gain (duplication) of the chromosomal region 14q11.2q24.2 arr[GRCh37] 14q11.2q24.2(20511672_70922704)x3) containing 437 genes was determined. These findings are suggestive of a chromosome translocation involving the chromosomes X and 14.

The infant cried immediately after birth and required only the initial steps of resuscitation. The Apgar scores were eight and nine at one and 10 minutes, respectively. Birthweight was 2270 grams (below fifth percentile), the length was 48 cm (25th centile), and head circumference was 32 cm (below fifth percentile).

Clinical examination showed a neonate with no apparent dysmorphic features with mild tachypnea (respiratory rate: 65 breaths per minute) and tachycardia (heart rate: 170 beats per minute). The patient's arterial blood gases report was pH-7.30, PaCO2 47 mmHg, base deficit -2 (BE-2); blood pressure was 62/40 mmHg, and oxygen saturation was 90% at room air. She had vesicular breath sounds with few fine basal crepitations. The first and second heart sounds were normal intensity, but with a systolic murmur grade three of six with maximal intensity at the left lower sternal edge. 

**Figure 1:**
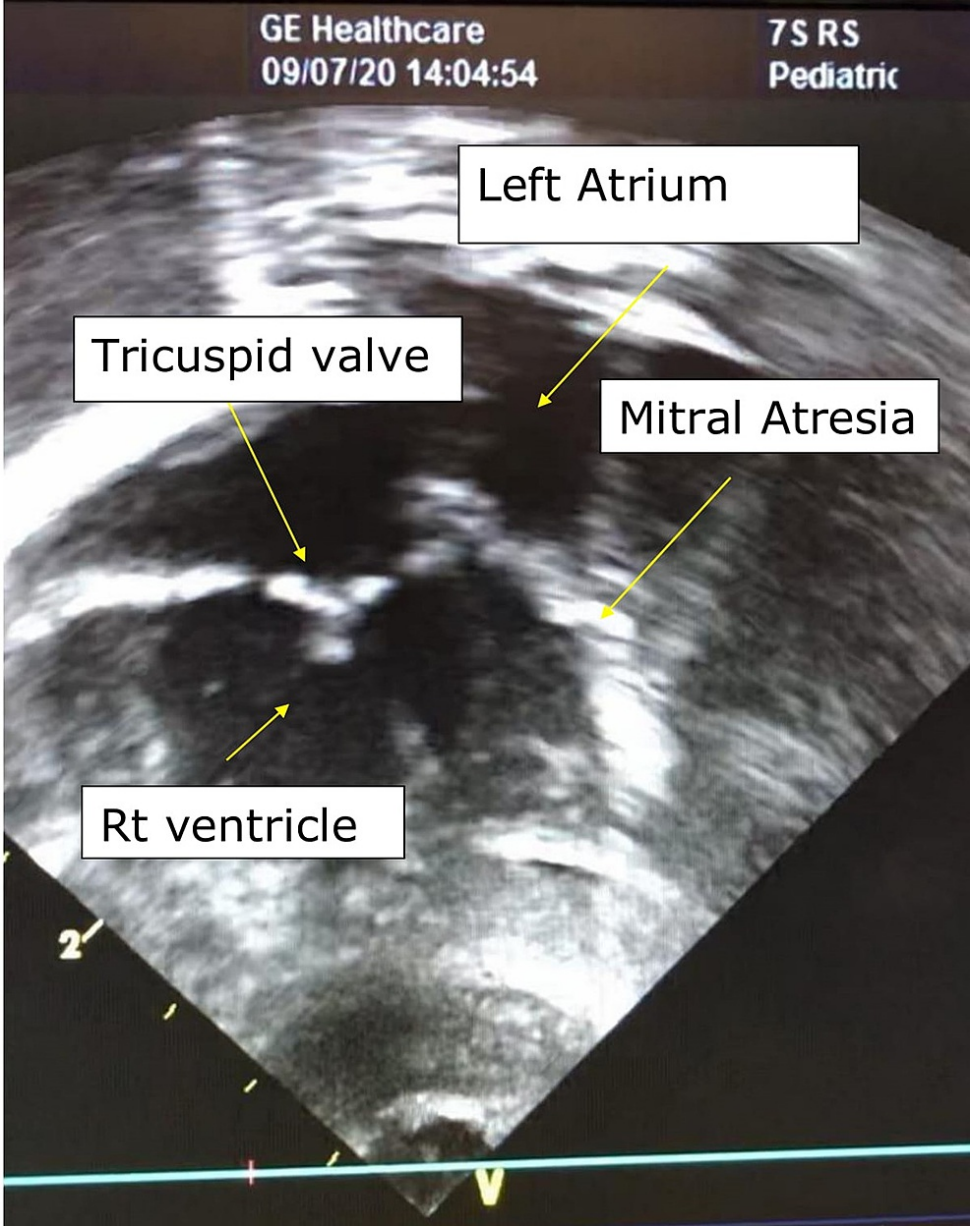
Echocardiography findings - apical four-chamber view shows atretic mitral valve and severe hypoplastic left ventricle

**Figure 2:**
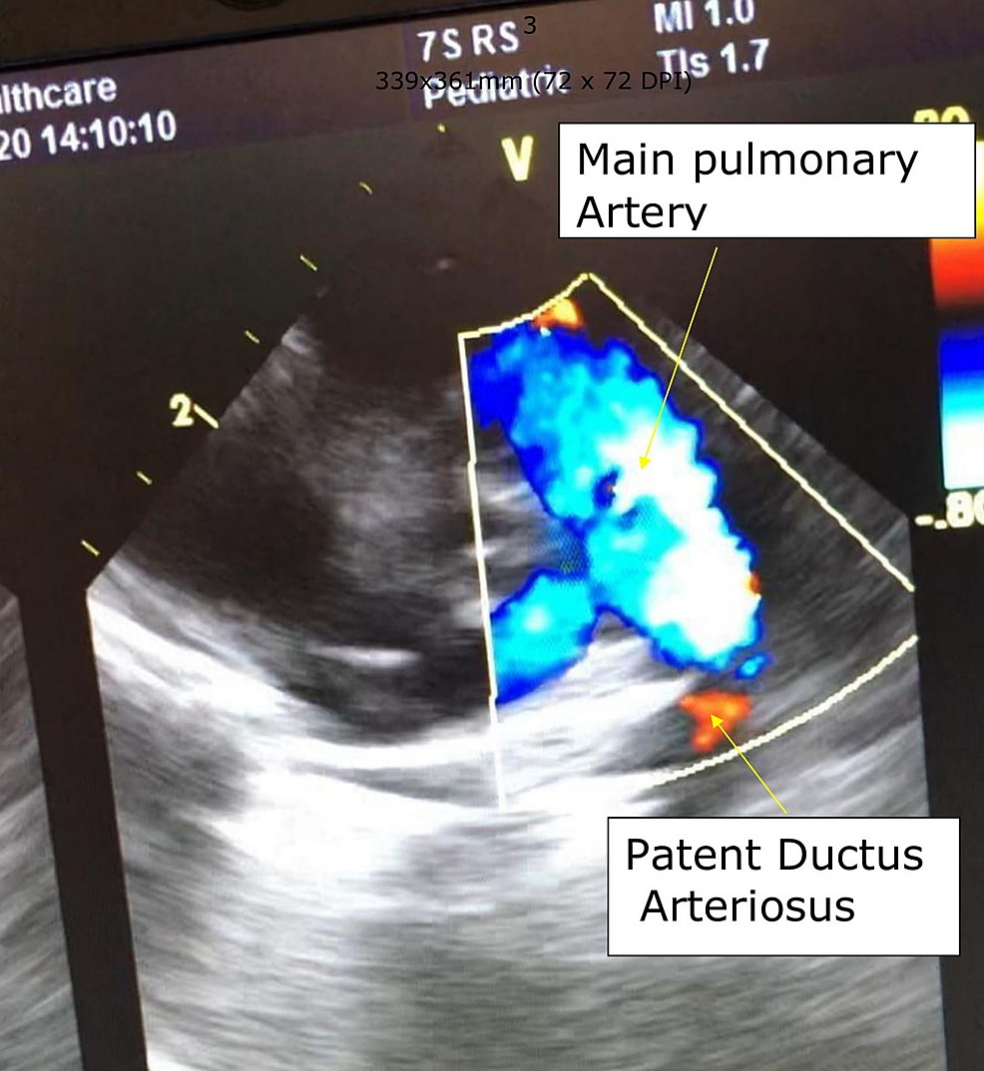
Short axis view shows main pulmonary artery and ductus arteriosus supplying the distal part of arch of the aorta

The postnatal echocardiography report confirmed the diagnosis of HLHS with the hypoplastic left ventricle, mitral atresia, aortic atresia, and moderate tricuspid regurgitation (Figures 1, 2).

The neonate was started on prostaglandin infusion treatment. Physical examination of other systems on admission was normal with stable hemodynamic values. Laboratory blood tests showed normal ranges of white blood cells, hemoglobin, and platelet counts. Abdomen ultrasonography and magnetic resonance imaging of the brain were unremarkable. A patient was diagnosed to have HLHS with partial monosomy X (Turner syndrome) and partial trisomy 14 (14q11.2 microduplication syndrome). We explained to parents the expected poor outcome of the management option of such cardiac defects; as a result, parents accepted the option of comfort care. The patient passed away on the seventh day of life.

## Discussion

The association of the HLHS with Turner syndrome (TS) was reported to be 13.2 %; though, only 2.5% of HLHS cases presented with TS [9,10]. The survival rate for infants with HLHS following the Norwood procedure at five years and 10 years can reach more than 65% and 55%, respectively [11]. However, the TS diagnosis in the patients with HLHS increased the mortality rate up to 80% [8,10,12]. Several other genetic disorders like Holt-Oram, Noonan syndrome, trisomy 21, trisomy 13, and trisomy 18 may also coexist with HLHS [13,14].

Chromosomal microdeletions and duplications appear to be another risk factor for the HLHS and can occur in up to 10% [15]. Microduplication syndrome 14q11.2 is a rare chromosomal aberrations condition characterized by hypotonia, mental retardation, developmental delay, and subtle dysmorphic craniofacial features [16].

Complete trisomy 14 has a high rate of fetal mortality, while live born chance increased with the mosaic type of trisomy 14 [17]. Çetin et al. described a case of 14q11.2 microduplication with West syndrome (infantile spasms, hypsarrhythmia, and intellectual disability) [18]. Other manifestations of 14q11.2 microduplication include microcephaly, behavior disturbance, obesity, and speech delay [19].

Turner phenotype is very variable, even in cases with a similar karyotype, though there are some common presentations like gonadal dysgenesis, skeletal malformations, and short stature. Furthermore, dysmorphic craniofacial features are not essential in neither 14q11.2 microduplication syndrome nor Turner syndrome [20,21]. Some cases, because of minimal clinical features and normal stature, will be diagnosed in adolescence as a part of a fertility assessment due to premature ovarian failure [22].

Leppig and Disteche reported that only cases with rings or fragments derived from the X chromosome would have a more severe phenotype that also depends on size, origin, the status of the X inactivation, and genes affected by copy number variations [23]. The absence of dysmorphic features in the presented case highlights the need for a genetic investigation in all patients with HLHS, even in non-dysmorphic neonates. Some authors mentioned the importance of CHD8 and SUPT16H genes in the pathogenesis of 14q11.2 microduplication syndrome [16,19]. Both CHD8 and SUPT16H genes are within the chromosomal region 14q11.2q 24.2 found in our case. 

Pathogenic variants in the MYH6 gene are a well-known cause of hypertrophic cardiomyopathy and dilated cardiomyopathy (MIM # 613251 and MIM # 613252). Pathogenic variants in the MYH6 gene also can cause structural heart abnormality, especially atrial septal defect [24,25]. Moreover, Theis et al. described heterozygosity for recessive MYH6 mutations in cases with HLHS that implicate a common molecular source for the latent myopathy of both ventricles [26]. Min-Su-Kim et al suggest that fetal heart development depends on proper blood flow. Furthermore, hemodynamic changes ensued due to the sarcomere disorganization may affect the development of the left ventricle [27]. Association of myosin-6 variants in patients with HLHS decreased cardiac transplant-free survival rate comparing with HLHS without myosin-6 variants [27,28]. The MYH6 gene is duplicated in our patient because it is located within the chromosomal region 14q11.2q 24.2.

Clinical manifestations and degree of mental retardation of 14q11.2 microduplication syndrome depend on the duplication size, which varies from small (e.g., 35 kilobases) to large size (e.g.,50 megabases). The presence of duplication and deletion in the case indicates these abnormalities may happen from a balanced translocation in one of the parents, encouraging us to send chromosomal analysis for parents to prove it.

Although HLHS association with Turner syndrome is well known, 14q11.2 microduplication with Turner syndrome and HLHS was not reported. Assumed the possibility of genetic heterogeneity of HLHS, antenatal genetic work-up should be standard in these cases.

## Conclusions

HLHS is one of the common cardiac defects that can be detected on prenatal sonography. Timely provided prenatal ultrasonography and prompt genetic investigation for each case of HLHS may reveal the natural origin of this complex congenital heart disease that is essential for determining the optimal decision-interventions options for physicians and parents.
